# Clinical diagnostic model for sciatica developed in primary care patients with low back-related leg pain

**DOI:** 10.1371/journal.pone.0191852

**Published:** 2018-04-05

**Authors:** Siobhán Stynes, Kika Konstantinou, Reuben Ogollah, Elaine M. Hay, Kate M. Dunn

**Affiliations:** Arthritis Research UK Primary Care Centre, Research Institute for Primary Care & Health Sciences, Keele University, Keele, Staffordshire, United Kingdom; University of Umeå, SWEDEN

## Abstract

**Background:**

Identification of sciatica may assist timely management but can be challenging in clinical practice. Diagnostic models to identify sciatica have mainly been developed in secondary care settings with conflicting reference standard selection. This study explores the challenges of reference standard selection and aims to ascertain which combination of clinical assessment items best identify sciatica in people seeking primary healthcare.

**Methods:**

Data on 394 low back-related leg pain consulters were analysed. Potential sciatica indicators were seven clinical assessment items. Two reference standards were used: (i) high confidence sciatica clinical diagnosis; (ii) high confidence sciatica clinical diagnosis with confirmatory magnetic resonance imaging findings. Multivariable logistic regression models were produced for both reference standards. A tool predicting sciatica diagnosis in low back-related leg pain was derived. Latent class modelling explored the validity of the reference standard.

**Results:**

Model (i) retained five items; model (ii) retained six items. Four items remained in both models: below knee pain, leg pain worse than back pain, positive neural tension tests and neurological deficit. Model (i) was well calibrated (p = 0.18), discrimination was area under the receiver operating characteristic curve (AUC) 0.95 (95% CI 0.93, 0.98). Model (ii) showed good discrimination (AUC 0.82; 0.78, 0.86) but poor calibration (p = 0.004). Bootstrapping revealed minimal overfitting in both models. Agreement between the two latent classes and clinical diagnosis groups defined by model (i) was substantial, and fair for model (ii).

**Conclusion:**

Four clinical assessment items were common in both reference standard definitions of sciatica. A simple scoring tool for identifying sciatica was developed. These criteria could be used clinically and in research to improve accuracy of identification of this subgroup of back pain patients.

## Introduction

Approximately two thirds of patients with low back pain (LBP) also report leg pain [1,2]. Once the possibility of serious spinal pathology (‘red flags’) or other non-spinal reasons for the leg pain are ruled out, the differential diagnosis is between leg pain due to nerve root involvement (sciatica) or non-specific referred pain from other structures in the low back. Making this diagnostic decision is recognised as difficult [3] and clinicians can disagree on diagnosis[4]. It is not always feasible or necessary to make specific diagnoses in a primary care setting (disc herniation, spinal stenosis) but early identification and differentiation of symptoms of low back-related leg pain (LBLP) (sciatica versus referred leg pain) are important for communicating likely diagnosis and prognosis to patients, formulating treatment plans and guiding the need for timely referrals to specialist services.

There is no universally agreed definition or clinical description of sciatica [5–7]. This has led to variability, limiting the generalisability of study findings in this subgroup of LBP patients, as the same disease entity may not be evaluated across studies. Individual items from history [8] and physical examination [9–11] have mostly shown poor diagnostic performance for identifying sciatica when compared to findings from MRI or surgery. Combining clinical assessment items is recommended to improve diagnostic performance [9,12]. In primary care, and other settings, clinicians assessing LBP patients integrate several patient characteristics and symptoms to form a diagnosis. Diagnosis in this case is therefore inherently multivariable [13]. Diagnostic models are tools that combine predictors to estimate the probability that a condition of interest is present in an individual with a certain predictor profile [14].

Previously published diagnostic models for LBLP [15–20] have variable methods of reference standard, predictor selection, and patient settings (primary care versus secondary care). Magnetic resonance imaging (MRI) is often used as the reference standard, but can result in misclassification, as positive MRI findings are found in asymptomatic individuals [21], and patients with nerve root symptoms may have normal MRIs [10]. In the absence of a well-accepted reference standard, expert clinical opinion may be an appropriate reference standard for diagnosis, providing it is reasonably reliable [22]. When clinicians diagnose LBLP with high levels of diagnostic confidence, reliability indexes are high (4).

To date, there is no consensus on what cluster of items best identify sciatica and there is no agreed gold standard for diagnosing sciatica. This study aims to identify the combination of items from clinical assessment that best identify sciatica in primary care consulters with LBLP, and to develop a diagnostic prediction model. To explore the challenges of reference standard selection for the diagnosis of sciatica, two reference standards (clinical diagnosis +/- MRI) were compared. Guidelines for Transparent Reporting of a multivariable model for Individual Prognosis or Diagnosis (The Tripod statement) [23] were followed.

## Materials and methods

### Ethics statement

All participants in the ATLAS study provided written consent to take part in the study, by signing a consent form in the presence of a trained research nurse, at the ATLAS research clinics. Ethical approval for the ATLAS study, including the consent procedure, was granted by the South Birmingham Research Ethics committee, reference number 10/H1207/82.

### Source of data and participants

Data from primary care consulters with LBLP taking part in the ATLAS (**A**ssessment and **T**reatment of **L**eg pain **A**ssociated with the **S**pine) observational cohort study [24] was analysed. Ethical approval for the ATLAS study was granted by the South Birmingham Research Ethics committee, reference number 10/H1207/82.

As part of the ATLAS study, patients completed questionnaires, underwent a standardised clinical assessment by one of seven musculoskeletal physiotherapists, and had a lumbar spine MRI within two weeks of their assessment (providing there were no clinical contraindications to the procedure). At the end of the clinical assessment, physiotherapists documented (i) a diagnosis of either sciatica or referred leg pain (ii) confidence (0–100%) in their diagnosis. Clinicians made their diagnosis based on information from history and physical examination findings only, the MRI findings were not part of the diagnostic process. For the purposes of the study, the term sciatica signifies spinal nerve root involvement. The MRI scans were scored by a senior consultant musculoskeletal radiologist, blind to any clinical information about the patient other than that the patient had LBLP (not specifying which leg). The radiologist provided a clinical report indicating definite, possible or absence of nerve root compression.

### Outcome

The outcome of interest in this study was a diagnosis of sciatica. Two reference standards were chosen for the diagnostic model:

Model (i): High confidence (≥ 80%) sciatica clinical diagnosis

Diagnosis of sciatica for model (i) reference standard was when the clinician documented the presence of leg pain was due to sciatica and they were ≥ 80% confident in their diagnosis. A cut off point of ≥ 80% diagnostic confidence was used because at this criterion, reliability among clinicians diagnosing LBLP improves considerably [4].

Model (ii): High confidence (≥ 80%) sciatica clinical diagnosis with confirmatory MRI findings

The second reference standard combined the clinician’s diagnosis with MRI findings (possible/definite) of nerve root compression. Using clinical diagnosis alone as a reference standard may leave diagnosis open to incorporation bias as the reference standard is not blind to knowledge of the predictors under consideration [25].

### Predictors

Nine candidate predictors were a priori chosen for potential inclusion in the diagnostic model from the larger set of available self-report and clinical assessment findings. Predictor selection was guided by (a) expert consensus from a Delphi study on items from clinical assessment considered most important for distinguishing sciatica from non-specific leg pain in LBLP patients [26] and (b) items used in other multivariable diagnostic models shown to have acceptable diagnostic accuracy for identifying sciatica [15–20].

Small or zero frequencies identified within 2x2 table cells made logistic regression using one variable unfeasible (myotomes). The three tests of myotomes, reflexes and sensation were therefore combined in a clinically acceptable way [27] to one variable: ‘any deficit on neurological testing’. Seven predictors remained for selection in the multivariable model (S1 Table for predictor selection and their measurement level).

### Sample size

Secondary data from an existing cohort study was used (S1 Dataset) hence no formal power calculation was performed to determine the sample size for the diagnostic model analysis. The sample size was adequate to satisfy the recommended guide of 10 events per predictor [28] with nine initial predictors and 100 patients in the smallest outcome category (patients diagnosed with referred pain for model (i)).

### Statistical analysis

Univariable logistic regression analysis quantified the relationship between each individual predictor variable and the presence of sciatica based on both reference standards. Multiple logistic regression with backwards stepwise selection (p = 0.05) was performed using all a priori selected predictor variables. Complete case analysis was planned because all but one of the baseline predictors had no missing data. Contribution of each predictor variable within the final model was presented as beta coefficients and odds ratios (ORs) with 95% confidence intervals (CI).

Measures of calibration and discrimination assessed predictive performance of the models. Calibration was assessed graphically using the observed outcome plotted against the predicted probability of the outcome obtained from the fitted logistic regression model using the Lowess smoothing curve technique [29]. Perfect calibration shows a slope on the 45 degree line, hence deviation of the line from the diagonal indicates lack of calibration. The plot was supplemented with the Hosmer and Lemeshow goodness-of-fit test [27]. P ≥ 0.05 supports the goodness-of-fit.

Discrimination (the ability of the model to distinguish between those who do and do not have the sciatica diagnosis) was summarised using the area under the curve (AUC) of the receiver operating characteristic (ROC) curve. An AUC of 0.5 indicates no discrimination whereas AUC of 1.0 indicates perfect discrimination [27].

Internal validity of the final model was assessed using 1000 bias corrected bootstrap samples. An adjusted AUC was calculated for the bootstrapped model to reflect the discriminative performance of the internally validated model.

Characteristics of the population used in diagnostic modelling were compared to those excluded from analyses due to application of high confidence in diagnosis criteria, using descriptive statistics.

To address the issue of an imperfect reference standard, a probabilistic statistical alternative using latent class (LC) modelling was used [30]. This method specifies a model so that response probabilities of clinical assessment items used to model the classes can be derived without knowing the patient’s true classification (diagnosis) [31]. The technique identifies latent or underlying groups of patients based on their response to clinical assessment items, and circumvents the need for a reference standard. It is therefore a useful comparator to the other analyses. Each patient was reclassified according to a two solution latent class model. It is assumed that the two latent classes correspond to one class of patients in which the target condition is present and one class in which the target condition is absent [31]. Concordance between the clinical diagnosis (+/- MRI) groups and the two latent classes was calculated using percentage agreement and a kappa statistic.

MPlus v5 was used for LC modelling. SPSS v21 and Stata v13 were used for the diagnostic model and descriptive analyses.

### Scoring tool

A simplified scoring tool for the best performing model was derived, to give a LBLP patient their probability of having sciatica. Regression coefficients for each predictor in the final model were converted to whole numbers by dividing each item coefficient by the lowest value coefficient [13]. Scores were presented alongside their associated outcome probabilities [32].

### Sensitivity analysis

An additional multivariable logistic regression model was performed using MRI only as the reference standard so as to compare to models published in the literature that have used MRI findings only as reference standard. The log ORs, corresponding CIs and AUCs of this additional model was compared to the original two models.

## Results

### Participants

Of the 609 LBLP consulters who participated in the ATLAS study, 395 participants were included in the diagnostic model development analysis and LC modelling. Reasons for excluding patients from the diagnostic model analysis were (i) if clinician confidence in diagnosis (for either referred leg pain or sciatica) < 80% (n = 173), (ii) patients did not have an MRI scan (an additional 41 patients).

Table 1 displays characteristics of patients in the diagnostic model development sample (n = 395) and those not included in model building analyses (n = 214). The excluded group had a greater proportion of patients aged over 65 years (18% v 14%), higher proportion of females (68% v 60%), more patients with leg symptoms for over 3-months (42% v 33%) and more comorbidity (17% ≥ 2 comorbidities v 11%). Comparing clinical characteristics, a greater proportion of patients in the diagnostic model group had a positive cough/sneeze (25.8% v 12 .6%), leg pain worse than back pain (50.1% v 38.3%), neurological deficits (57.7% v 46.3%) and positive neural tension tests (60.8% v 44.4%).

**Table 1:** Characteristics of patients eligible and ineligible for diagnostic model development data.

	Diagnostic model cases	Excluded cases
	n = 395	n = 214
Age (years), mean (sd)	49.8 (13.9)	50.9 (13.9)
Age Categories		
18–34 years 35–44 years 45–54 years 55–64 years 65+ years	64 (16.2) 82 (20.8) 102 (25.8) 93 (23.5) 54 (13.7)	27 (12.6) 54 (25.2) 50 (23.4) 45 (21.0) 38 (17.8)
Gender: Female	237 (60.0)	146 (68.2)
BMI category: obese ^a^	160 (40.5)	88 (41.1)
Self-certified time off work or given sick note due to current episode ^b^	99 (40.9)	45 (37.2)
Back pain intensity [33], mean (sd) ^c^	5.5 (2.2)	5.7 (2.1)
Leg pain intensity [33], mean (sd) ^c^	5.3 (2.4)	5.1 (2.4)
RMDQ disability score (0–23) [34], mean (sd) ^d^	12.8 (5.7)	12.4 (5.8)
Duration of back symptoms		
Less than 6 weeks 6–12 weeks >3 months	132 (33.6) 94 (23.9) 167 (42.5)	86 (40.1) 32 (15.0) 96 (44.9)
Duration of leg symptoms		
Less than 6 weeks 6–12 weeks >3 months	165 (43.9) 85 (22.6) 126 (33.5)	86 (41.5) 35 (16.9) 86 (41.6)
General Health: Fair/poor	143 (36.3)	79 (36.9)
Two or more other health problems ^e^	43 (10.9)	37 (17.3)
Positive cough/sneeze	102 (25.8)	27 (12.6)
Below knee pain	278 (70.4)	152 (71.0)
Leg pain worse than back pain	198 (50.1)	82 (38.3)
Subjective sensory changes in leg	243 (61.5)	139 (65.0)
Neurological tests deficit (any positive test) Myotomes Dermatomes Reflexes	228 (57.7) 81 (20.5) 173 (43.8) 91 (23.0)	99 (46.3) 24 (11.2) 80 (37.4) 28 (13.3)
Neural tension tests (any positive test) Straight leg raise (SLR) Crossed SLR Slump Femoral nerve stretch	240 (60.8) 221 (55.9) 21 (5.3) 64 (16.2) 27 (6.8)	95 (44.4) 76 (35.5) 1 (0.5) 20 (9.3) 14 (6.5)
Positive MRI findings for nerve root compression	231 (58.5)	66 (30.8)
Clinical diagnosis; Stenosis	34 (8.6)	14 (6.5)

Abbreviations: sd, standard deviation; BMI, body mass index; RMDQ, Roland Morris Disability Questionnaire; MRI, magnetic resonance imaging.

All figures are frequencies (percentages) unless stated otherwise as mean (sd).

^a^ BMI obese category = 30 to 40^+^ kg/m^2^

^b^ Applicable to only those working n = 365

^c^ Pain intensity measured using the mean of three 0 to 10 numerical rating scales for least and usual back pain over the previous 2 weeks and current back pain intensity

^d^ Roland Morris Disability Questionnaire leg pain version with scores from 0–23 with higher scores indicating higher disability

^e^ The comorbidity health problems included chest problems, heart problems, raised blood pressure, diabetes, circulation problems in the legs

Of the 395 patients included in the analysis, 75% (n = 295) were diagnosed with sciatica using model (i) reference standard. Using model (ii) reference standard, where clinical diagnosis was corroborated by positive MRI findings, 51% (n = 200) were diagnosed with sciatica.

Class one identified by LC modelling had 244 patients, class two had 151 patients. The overall percentage agreement between the clinical diagnosis groups defined by model (i) and the two latent classes was 83%, with a kappa coefficient of 0.62 (95% CI 0.54, 0.70) indicating substantial agreement [35]. This suggests the clinical diagnosis reference standard was adequate. Comparing the two latent classes to groups diagnosed using high confidence clinical diagnosis and confirmatory MRI findings (model ii), showed agreement of 72% and kappa 0.43 (95% CI 0.35, 0.52) indicating moderate agreement.

### Model development

Following univariable analysis, all predictor variables were significantly associated with both reference standard outcomes (p<0.001) (Table 2). The ORs for model (i) were all higher than model (ii). The greatest strength of association with model (i) diagnosis was ‘positive neural tension tests’ with very high ORs of 31.9. For model (ii), ‘leg pain worse than back pain’ had the highest association with the diagnosis (6.1; 3.9, 9.4).

**Table 2:** Univariable associations between predictor variables and outcomes.

**Model (i) reference standard: high confidence (≥80%) clinical diagnosis**				
**Item variable**	**Patients with sciatica**	**Patients without sciatica**	**Univariable odds ratio**	**95% CI**
Subjective sensory changes	161 (55)	12 (12)	3.9	2.5, 6.3
Below knee pain	250 (85)	28 (28)	14.3	8.3, 24.5
Leg pain worse than back pain	183 (62)	15 (15)	9.3	5.1, 16.8
Cough sneeze	95 (32)	7 (7)	1.5	1.4, 1.7
Intensity leg pain mean (sd)	5.8 (2.3)	3.6 (2.0)	6.3	2.8, 14.1
Neural tension tests	230 (78)	10 (10)	31.9	15.7, 64.7
Neurological deficit	212 (72)	16 (16)	13.4	7.4, 24.2
**Model (ii) reference standard: high confidence (≥80%) clinical diagnosis plus MRI**				
Subjective sensory changes	110 (55)	63 (32)	1.9	1.3, 2.9
Below knee pain	173 (87)	105 (54)	5.5	3.4, 9.0
Leg pain worse than back pain	142 (71)	56 (29)	6.1	3.9, 9.4
Cough sneeze	79 (40)	23 (12)	1.4	1.3, 1.5
Intensity leg pain mean (sd)	6.1 (2.3)	4.4 (2.2)	4.9	2.9, 8.2
Neural Tension tests	157 (79)	83 (43)	4.9	3.2, 7.7
Neurological deficit	149 (75)	79 (41)	4.3	2.8, 6.6

Abbreviations: CI, confidence intervals; sd, standard deviation; NS, non-significant at p ≤ 0.05; MRI, magnetic resonance imaging

### Model specification

Multivariable analysis was performed on 394 participants since one variable had missing data on one patient. Results are presented in Table 3. The clinical diagnosis reference standard model (i) produced a final model with five items (p<0.05). Positive cough/sneeze and intensity of leg pain were eliminated. Six items were retained in model (ii), only subjective sensory changes was eliminated. Four items were retained in both models: below knee pain; leg pain worse than back pain; positive neural tension tests; neurological deficit. The ORs for model (i) were all higher than model (ii).

**Table 3:** Multivariable associations between the clinical assessment items and sciatica for model (i) and model (ii).

Item variable	Model (i)		Model (ii)		
	Beta	OR (95% CI)		Beta	OR (95% CI)
Subjective sensory changes	0.98	2.66 (1.20, 5.90)		NS	NS
Below knee pain	1.83	6.25 (2.80, 13.94)		0.76	2.13 (1.19, 3.83)
Leg pain worse than back pain	1.52	4.55 (1.89, 10.99)		1.08	2.94 (1.77, 4.89)
Leg pain intensity	NS	NS		0.14	1.15 (1.03, 1.29)
Positive cough / sneeze	NS	NS		0.92	2.50 (1.34, 4.65)
Neural Tension tests	3.07	21.63 (9.00,51.97)		0.56	1.76 (1.03, 3.00)
Neurological deficit	2.14	8.50 (3.80,19.01)		1.04	2.81 (1.69, 4.69)
Intercept	-3.25			-2.98	
**AUC**		0.95 (0.93, 0.98)			0.82 (0.78, 0.86)

Abbreviations: OR, odds ratios; CI, confidence intervals; AUC, area under the receiver operating characteristic curve; NS, non-significant at p≤ 0.05

Model (i): Confidence ≥80% sciatica clinical diagnosis

Model (ii): Confidence ≥80% sciatica clinical diagnosis plus confirmatory magnetic resonance imaging (MRI) findings

The predicted probability of sciatica can be calculated using the following formulae

Model (i): Probability (sciatica^+^) = 1/ 1+exp -[-3.25 + (subjective sensory changes x 0.98) + (below knee pain x 1.83) + (leg pain worse than back pain x 1.52) + (neural tension x 3.07) + (neurological deficit x 2.14)].

Model (ii): Probability (sciatica^+^) = 1/ 1+exp -[-2.98 + (below knee pain x0.76) + (leg pain worse than back pain x 1.08) + (intensity leg pain x 0.14) + (positive cough/sneeze x 0.92) + (neural tension x 0.56) + (neurological deficit x 1.04)]

### Model performance

**Fig 1:**
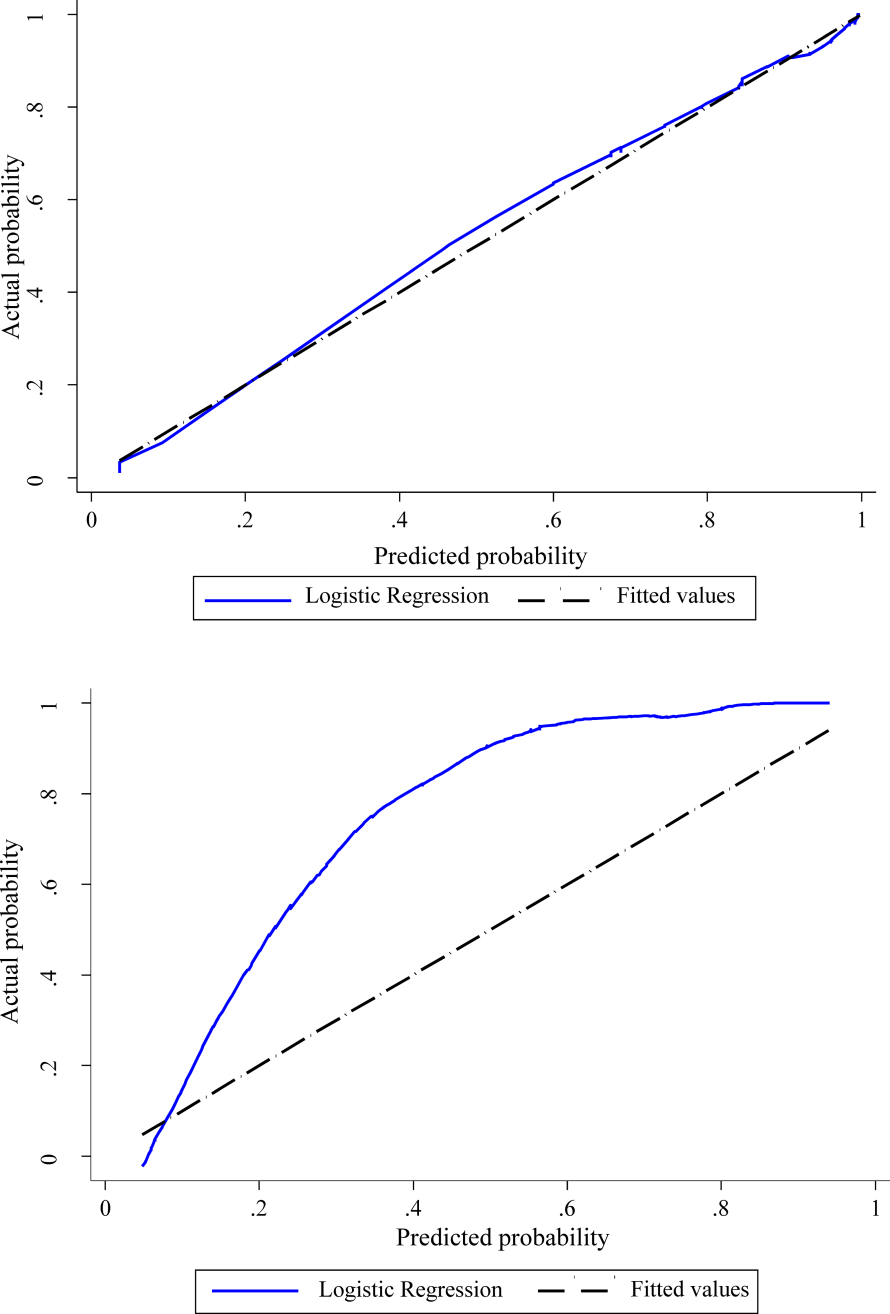
Calibration plot for model (i) and model (ii). Model (i) calibration shown in upper graph, model (ii) calibration shown in lower graph. The solid line is a smoothed curve that represents an estimate of the relation between the predicted and the observed probability of sciatica diagnosis. Ideally this line fits the dotted line that represents perfect calibration.

The shape of the slope on the calibration plots show that model (i) is well calibrated and model (ii) less well calibrated (Fig 1). The Hosmer and Lemeshow statistical test for the observed data for model (i) supported the goodness-of-fit of the model (χ^2^ = 11.4, p = 0.18) whereas model (ii) showed poor calibration (χ^2^ = 22.4 p = 0.004).

Discrimination was almost perfect for model (i) (AUC 0.95, 95% CI 0.93, 0.98) and excellent for model (ii) (AUC 0.82, CI 0.78, 0.86). Adjusted AUCs for both models were unaltered following bootstrapping.

A simple scoring method, for the better performing model (i), was developed by converting the beta coefficient values into whole numbers. A total score of 10 could be achieved (Table 4). The corresponding predicted probability of sciatica for each sum score was calculated. Using this clinical diagnostic model (with high confidence clinical diagnosis as the reference standard), a threshold score of 5 or above suggests high likelihood of being diagnosed with sciatica (at least 83%). Using coordinates from the ROC curve, at this threshold, the model has sensitivity of 0.85 and specificity of 0.88.

**Table 4:** Scoring tool based on model (i) for clinical assessment items and corresponding predicted probability of sciatica.

**Variable in the model**	**Does the patient:**									**Score**	
Subjective sensory changes	Report any pins and needles or numbness in the involved lower limb									1	
Below knee pain	Report pain below the knee									2	
Leg pain worse than back pain	Report that their leg pain is worse than their back pain									2	
Neural Tension tests	Have a positive straight leg raise test and/or femoral nerve test and/or slump test									3	
Neurological deficit	Have any myotome/ reflex or sensory deficit in the involved lower limb									2	
	**Sum Score**									….	
**Sum Score**	**0**	**1**	**2**	**3**	**4**	**5**	**6**	**7**	**8**	**9**	**10**
N	36	19	19	20	21	47	29	41	61	25	76
Observed Sciatica (%)	3	11	11	50	67	85	86	100	97	100	100
Mean predicted probability of Sciatica (%)	**4**	**9**	**19**	**42**	**63**	**83**	**93**	**96**	**99**	**100**	**100**

### Sensitivity analyses

When MRI only was the reference standard, the predictors remaining in the model were leg pain worse than back pain (OR 2.4, CI 1.6, 3.4), intensity of leg pain (OR 1.1, CI 1.0, 1.2), positive cough/sneeze (OR 2.0, CI 1.3, 3.2) and neurological deficit (OR 1.7, CI 1.2, 2.4). Positive neural tension tests, below knee pain and subjective sensory changes were not in the final model. This model had the lowest AUC (0.70, CI 0.65, 0.74).

## Discussion

This study ascertained the items from clinical assessment that best identify sciatica in primary care consulters with LBLP. In the absence of a gold standard for diagnosing sciatica, two reference standards were compared. Model (i), using high confidence in clinical diagnosis as a reference standard, retained five items and had almost perfect calibration and discrimination. Model (ii), with the addition of confirmatory MRI in the reference standard, retained six items and showed good discrimination but poor calibration. Bootstrapping revealed minimal overfitting in both models.

The predictors that were retained in both models are unsurprising from a clinical perspective. “Pain below the knee” is commonly considered a proxy for sciatica [36] and other diagnostic models report its association with nerve root involvement defined by either clinical diagnosis [17] or MRI [19]. The “leg pain worse than back pain” item performed strongly in both models and two previous diagnostic models reported its association with sciatica [16,17]. However, it has received less attention in the literature, for example as an eligibility criterion for selecting sciatica patients in intervention studies [37]. In clinical practice, cough/sneeze/strain reproducing leg pain is considered indicative of sciatica. In this analysis “positive cough/sneeze” was significant in model (ii), similar to associations seen in other models using MRI findings as the reference standard [16,18], but not significant in model (i). Self-report symptoms of weakness or numbness have previously shown minimal association with MRI findings of nerve root compression [19], similar to our findings for model (ii). Neurological deficit was associated with sciatica in both models. “Positive neural tension tests” remained in both models but with considerable difference in the magnitude of the ORs. The model including MRI in the reference standard gives much less weight to the association between positive neural tension and sciatica (OR 1.8). When MRI findings only were used as the reference standard, positive neural tension was not predictive of sciatica diagnosis, similar to a previously published model which used MRI as the reference standard [16]. Clinically and in the literature it is recognised as a diagnostic criterion for sciatica [6]. It is suggested that neural tension tests may cause pain due to chemical mediators irritating the nerve root but not generating detectable signal on MRI [19].

Different choice of patient population and reference standards (MRI versus clinical diagnosis) limits readers’ ability to compare diagnostic models for sciatica. Four models in the literature have used MRI as reference standard [15,16,18,19]; two of these included only self-report items as predictors [15,19]. One model used clinical diagnosis as the reference standard [17] and the oldest published model used mylegrophy [20]. Three of the six models are based on patients in secondary care settings [15,19,20], with potentially more severe presentations than those from primary care settings [16–18].

Performance measures are not always reported [18–20] which makes it difficult to compare models. In a model that used nerve root compression on MRI as a reference standard; gender and sensory loss remained significant predictors, but performance was poor (AUC 0.65) [15]. History items alone were used to develop the model and the population was a highly selected group with severe sciatica. Items identified by Vroomen et al. [16], to be associated with nerve root compression defined by MRI, performed well (AUC 0.80) for demographic (age and gender) and history domains (spasmodic pain, pain worse in leg than back, pain in a dermatomal distribution, positive cough/sneeze). Their model performance improved slightly (AUC 0.83) when physical examination items were added (restricted forward bending, myotome weakness). External validation of the history items in a different data set resulted in a much lower AUC of 0.58 [15]. Using a similar reference standard and population setting to the study in this report, Konstantinou et al. [17] also found pain below knee, leg pain worse than back pain and feeling of numbness or pins and needles to be associated with the clinical diagnosis of sciatica. The authors acknowledge that not including clinical examination items may explain their models’ performance (AUC 0.72 for only definite cases of sciatica; AUC 0.74 for definite and possible cases of sciatica indicated by clinical diagnosis).

### Limitations

As there is no gold standard for diagnosing sciatica, selection of a reference standard is always a challenge. In this study, for Model (i), expert clinical opinion was chosen as a reference standard, which is considered in some circumstances appropriate in the development of diagnostic criteria in the absence of a gold standard [22]. It also reflects current practice in primary care when in the majority of cases, diagnosis and initial management plans are put into place without access to imaging, at least initially. Patients excluded from the analysis were cases where clinicians indicated low diagnostic confidence, irrespective of either a referred leg pain or sciatica diagnosis. A reliability study, nested in this cohort, showed good reliability on diagnosis of LBLP when clinician confidence is high (at least 80%) [4]. Diagnostic uncertainty is a clinical reality as sometimes a return visit from the patient is needed to further confirm or explore diagnosis. All patients received an MRI scan as part of this research study and patients were not selected for inclusion in the study based on the results of this scan.

The clinicians unavoidably used information from the assessment predictor variables to make their diagnosis; this contributes to incorporation bias and potentially inflates accuracy estimates [38,39]. Ideally the reference standard and the predictors should be independent of one another to avoid inflation of accuracy estimates [38,39].

A second reference standard was chosen which combined confirmatory MRI findings with the high confidence clinical diagnosis, in order to address to some extent the issue of incorporation bias.

Alternative approaches to deal with an “imperfect reference standard” include using a combination of reference standards in a sequential manner to diagnose patients [38]. For example firstly interpreting clinical information, then, if needed, combining this information with further diagnostic tests (e.g. MRI). Another recommended means of limiting bias with reference standard selection is the use of consensus so more than two assessors agree on a diagnosis [38]. However, both these methods can result in selection bias as the “easier to identify” cases are selected therefore losing the heterogeneity of patients seen in normal clinical life.

Using MRI only as a reference standard, which allows the reference standard and predictors to be independent of each other, produced the lowest performance index (AUC 0.70) and did not retain the predictors “pain below the knee” and “positive neural tension tests”. Excluding these variables is at odds with clinical opinion and evidence in the literature, and reflects the mismatch seen in studies between clinical presentation and MRI findings [40].

The latent class analysis was performed to classify patients into two groups without the need for a reference standard. The two class solution showed good concordance with the groups defined as referred pain and sciatica according to clinical diagnosis reference standard, supporting the validity of clinical diagnosis for use as a reference standard.

Stepwise regression is an automated process and using too many variables and removal of variables that may be important are some of the recognised limitations of the technique [13]. For this model the number of predictors was not excessive in relation to the sample size [28] and the backwards approach to predictor selection allows the model to be assessed as the variables are removed sequentially.

The choice of predictor selection for this model was primarily based on previous consensus work on items from clinical assessment that contribute most to the diagnosis of LBLP due to sciatica [26]. The primary care setting of this study helps to limit the issue of selection bias seen in other diagnostic studies where patients are selected from secondary care settings and have more severe symptoms. Assessors who participated in this study were all experienced physiotherapists and underwent training to enhance standardisation of the data collection and diagnostic decisions. It could be argued that diagnostic accuracy may be better among other healthcare professionals or medically trained clinicians. However previous work showed that agreement among clinicians is similar between physiotherapists and other healthcare professionals when diagnosing sciatica [4].

### Conclusion

This study used information from clinical assessment to estimate the likelihood of sciatica in patients with LBLP presenting in the primary care setting. It is the first study to explore the considerable challenges, implications and sources of bias inherent with reference standard selection in identifying sciatica, and to compare models with different reference standards. A clear cluster of items was found which consistently identified sciatica: pain below the knee, leg pain worse than back pain, positive neural tension and neurological deficit. A simple scoring tool was developed which could prove useful to clinicians and researchers wishing to support their clinical judgement regarding the probability of whether a patient’s leg pain is sciatica. In research settings, the tool could enable more optimum identification of a homogenous group.

## Supporting information

S1 Table Predictor selection and their measurement level. (DOCX)

S1 Dataset (XLSX)
