# Supplementary material for: Clinical diagnostic model for sciatica developed in primary care patients with low back-related leg pain
Source: PLoS One. 2018 Apr 5;13(4):e0191852. doi: 10.1371/journal.pone.0191852 (PMC5886387; doi:10.1371/journal.pone.0191852)
Supplement: S1 Table — (DOCX) [file pone.0191852.s001.docx]

**S1 Table Description of predictors selected for the multivariable diagnostic model for sciatica**

**Self-report/history items**

Subjective sensory changes (yes/no): A positive response was recorded if patient reported they had noticed symptoms such as numbness, pins and needles or tingling in their leg.

Below knee pain (yes/no): A positive response was recorded if the assessing physiotherapist marked any areas of pain below the knee on the body chart manikin; this could include areas of pins and needles or numbness below the knee.

Leg pain worse than back pain (yes/no): A positive response was recorded if the patient reported that their leg pain is worse/ or bothers them more than their back pain.

Leg pain intensity (0-10): This was measured using the mean of three 0 to 10 numerical rating scales for ‘least’ and usual’ leg pain over the previous two weeks and ‘current’ leg pain.

Positive cough or sneeze (yes/no): A positive response was recorded if patient’s leg pain (or on occasion buttock pain) is reproduced or increased on coughing, sneezing or straining.

**Physical examination items**

Neurological deficit (yes/no) A deficit was described as any myotome, reflex or sensory deficit:

Myotome deficit (yes/no). A deficit was defined as less than normal muscle strength (grade 5/5 on Oxford manual muscle testing scale) in any of the tested myotome muscle groups of the symptomatic lower limb(s).

Reflex deficit (yes/no): A deficit was defined as either a reduced/significantly reduced or absent tendon reflex (at the knee or the ankle) in the symptomatic lower limb(s).

Sensory deficit (yes/no): A deficit was defined as either a reduced, significantly reduced or absent (anaesthesia) response to pin-prick testing of dermatomal distribution areas in the symptomatic lower limb(s).

Positive neural tension tests (yes/no): Straight leg raise (SLR) test, crossed SLR, femoral nerve stretch test, slump test. A positive neural tension test was defined as reproduction of the patient’s leg pain ring performance of any of the neural tension tests.
